# Interhospital variation in surgical treatment of screen-detected breast cancer in the South of the Netherlands

**DOI:** 10.1016/j.breast.2025.103886

**Published:** 2025-01-20

**Authors:** Eline L. van der Veer, Angela M.P. Coolen, Adriana M.J. Bluekens, Manon I. Generaal, Robert-Jan Schipper, Wikke Setz-Pels, Dominique J.P. van Uden, Adri C. Voogd, Lucien E.M. Duijm

**Affiliations:** a Elisabeth TweeSteden Hospital, Hilvarenbeekse Weg 60, 5022, GC, Tilburg, Netherlands; b Erasmus Medical Centre, Dr. Molewaterplein 40, 3015, GD, Rotterdam, Netherlands; c Maastricht University, Department of Epidemiology, P. Debyelaan 25, 6229, HX, Maastricht, Netherlands; d Catharina Hospital, Michelangelolaan 2, 5623, EJ, Eindhoven, Netherlands; e Canisius Wilhelmina Hospital, Weg door Jonkerbos 100, 6532, SZ, Nijmegen, Netherlands

**Keywords:** Interhospital variations, Therapy, Breast cancer, Screening

## Abstract

**Background:**

The effectiveness of the Dutch breast cancer screening programme depends on the quality of the full trajectory, from the first screening to the final treatment of a screen-detected breast cancer. Interhospital variation in breast cancer treatment has been explored by several studies, however, not specifically in a screen-detected breast cancer population. The current study compares the treatment strategies of women with screen-detected breast cancer between hospitals in the South of the Netherlands.

**Methods:**

A total of 1450 women with screen-detected breast cancer, who participated in the Dutch screening programme between January 2009 and July 2019, were included in this retrospective analysis of a prospectively obtained database. Breast cancer treatment (i.e. preoperative MRI, neoadjuvant systemic therapy and type and outcomes of surgery) was compared between hospitals using multivariate analysis.

**Results:**

Statistically significant interhospital variation was observed in the use of preoperative MRI (range 20.8–35.8 %, p < 0.001), neoadjuvant systemic therapy (range 4.0–13.3 %, p < 0.001) and breast conserving surgery (range 70.0–87.1 %, p < 0.001). These differences persisted after adjustment for case-mix. In patients with invasive breast cancer treated by breast conserving surgery, the mean volume of the resection specimen ranged from 381 to 541 ml between hospitals (p < 0.001). However, this was not accompanied by significant differences in the percentage of patients with positive resection margins (range 2.9–5.7 %, p = 0.34).

**Conclusions:**

We observed significant interhospital variation in the management of women with screen-detected breast cancer. Quality assurance in screen-detected breast cancer may reduce these differences, but evolving breast cancer care and more personalised approaches should be accounted for.

## Highlights

• Treatment of screen-detected cancers (SDC) is subject to interhospital variation.
• Percentages of women undergoing preoperative breast MRI differed between hospitals.
• Variations in type of surgical treatment of invasive breast cancer were found.
• Variation in resection volume did not result in differences in tumour-free margins.
• Results suggest moderate guideline adherence, but may align with personalised care.

## Abbreviations

BCS: Breast conserving surgery
BI-RADS: Breast Imaging Reporting and Data System
B&R: Bloom & Richardson
DCIS: Ductal carcinoma in situ
HER2: Human Epidermal growth factor Receptor 2
MRI: Magnetic Resonance Imaging
NST: Neoadjuvant systemic therapy
SDC: Screen-detected cancer

## 1 Introduction

The Dutch breast cancer screening programme is a well-functioning, strictly regulated and monitored programme, providing free biennial screening mammography to asymptomatic women aged 50–75 years. Screen-detected breast cancers (SDC) are generally detected at an earlier stage, resulting in an improved prognosis [1,2].

To achieve maximum effectiveness of the breast cancer screening programme, it is essential that recall is followed by both an optimal clinical diagnostic work-up and, when necessary, therapeutic trajectory. It is plausible that substantial interhospital variation has a negative effect on quality of the breast cancer screening programme and general breast cancer care.

Once the diagnosis of breast cancer is confirmed, a treatment plan is drawn up by a multidisciplinary team. Treatment may involve surgery, adjuvant radiotherapy, (neo-)adjuvant chemotherapy, hormone therapy, HER2-targeted therapy, or a combination of these therapies. The type of treatment is dependent on tumour characteristics (i.e. size, histologic type, hormone receptor status, metastases) as well as patients’ age, health and preferences. [3].

Both national and international studies have been performed on interhospital variations regarding breast cancer therapy. Recently, a Dutch study group proposed 24 quality indicators for breast cancer treatment. They found medium interhospital variation (SD 5–15 %) in 12 of these 24 quality indicators, mainly regarding type of breast surgery and systemic treatment. Furthermore, large interhospital variations (SD ≥ 15 %) were reported in eight of 24 indicators, mostly regarding systemic treatment [4]. In other countries variations have also been observed regarding breast surgery and systemic therapy [5,6]. These studies included a mixed population of both symptomatic and asymptomatic women with breast cancer. To our knowledge, no studies have been performed specifically focussing on women with SDC. The latter can be regarded as a distinct population, as SDCs are generally smaller and less often metastasised to the lymph nodes or distant sites at diagnosis when compared to symptomatic breast cancers. Therefore, in the current study, the aim was to evaluate interhospital variations in breast cancer treatment in women with SDC.

## 2 Materials and methods

### 2.1 Study population

All women diagnosed with SDC between January 2009 and July 2019 were included. One of the screening radiologists (LD) collected data from radiological, pathological and clinical reports, as well as from a questionnaire. Women with a delay in their breast cancer confirmation of 12 months or more after their recall, were excluded from this study, to avoid including screened women who have worse outcomes due to disease progression during the extended diagnostic process.

Before participating in the Dutch screening programme, women are routinely asked for permission to use their data for scientific purposes. Three women did not consent and were therefore excluded. Ethical approval was waived by the Dutch Central Committee on Research involving Human Subjects (CCMO).

Subdivision of the study group was based on the hospitals to which women were referred for work-up after their screening recall. In our screening region in the South of the Netherlands there are six regional hospitals with a dedicated breast unit, where most of the diagnostic work-up and treatment of recalled women takes place. Incidentally, a recalled woman undergoes her diagnostic work-up and breast cancer therapy elsewhere, e.g. in a hospital located outside our screening region or even abroad. These women were analysed as a seventh group. Hospitals that had merged during the inclusion period are reported as one hospital.

### 2.2 Diagnostic work-up and treatment

We included the following diagnostic and therapeutic parameters: the preoperative confirmation of breast cancer, use of preoperative MRI, use of NST, final type of breast surgery, volume of the resection specimen and tumour presence at surgical specimen resection margins.

Preoperative confirmation of breast cancer was routinely obtained by percutaneous biopsy. In case of (repeated) equivocal findings at percutaneous biopsy, surgical biopsy was performed to exclude or confirm breast malignancy. In case a woman was diagnosed with multifocal, multicentric or bilateral breast cancer, only the largest tumour was included in our analysis. Tumour size was measured by the pathologist on the resection specimen. Tumour size was determined on imaging in women who abstained from surgery or who were physically unable to undergo surgery. In case of neoadjuvant systemic therapy (NST), tumour size was measured on imaging prior to the start of NST, usually on breast MRI. Volume of the resected specimen was determined using the formula for ellipsoid bodies. Tumour characteristics included histologic type, Bloom & Richardson grading [7], tumour and lymph node stage [8], tumour size, and hormone and HER2-receptor status. After pathological confirmation of a SDC, treatment is started as soon as possible. The participating hospitals all have to adhere to the same, most recent Dutch breast cancer guidelines [3,9,10].

Breast MRI may be performed prior to NST or surgery. According to both the Dutch and European guidelines, indications for pre-operative breast MRI include: 1) evaluation of the extent of the breast cancer when inconclusive or inconsistent on mammography and/or ultrasound, 2) evaluation of potential multifocal, multicentric or bilateral breast cancer (particularly in case of invasive lobular carcinoma), 3) evaluation of the extent of high grade DCIS, and 4) response evaluation in case of NST [[10], [11], [12], [9]].

NST included chemotherapy, hormonal therapy, HER2-targeted therapy, or a combination of these treatments. Breast surgery comprised either BCS or mastectomy. Abstaining from surgery was considered a treatment option as well. In case of oncoplastic surgery, the mean volume of the resection specimen could not be measured, and these breast cancers were therefore excluded from analysis.

A surgical margin was defined as tumour positive when cancer was present in at least 4 mm of the inked resection plane [13]. For the current analysis, a merely focal positive resection margin (tumour present in less than 4 mm of the inked resection plane) was considered a negative resection margin, as these women usually received a higher dose of adjuvant radiotherapy (boost) rather than repeated surgery [3].

### 2.3 Statistical analysis

Analyses were performed using Statistical Package for Social Science 27.0 (SPSS Inc., IBM, Chicago, IL). As the screening BI-RADS score was only added to the database since 2014, we lacked data on this characteristic for the time period 2009–2014. Patient and tumour characteristics were described using descriptive statistics. Significance of the comparison (p-values) of the baseline characteristics are not presented. These values could be misleading in large numbers of patients, and these differences between hospitals in baseline characteristics, usually referred to as case-mix, are only used for correction in the multivariate analyses. Chi-square tests were performed to compare interhospital differences. Logistic regression analyses were performed to analyse therapeutic differences between hospitals while correcting for confounders, using backward elimination. All statistical tests were two-sided. P-values less than 0.05 were considered statistically significant.

## 3 Results

### 3.1 Study population

**Fig. 1:**
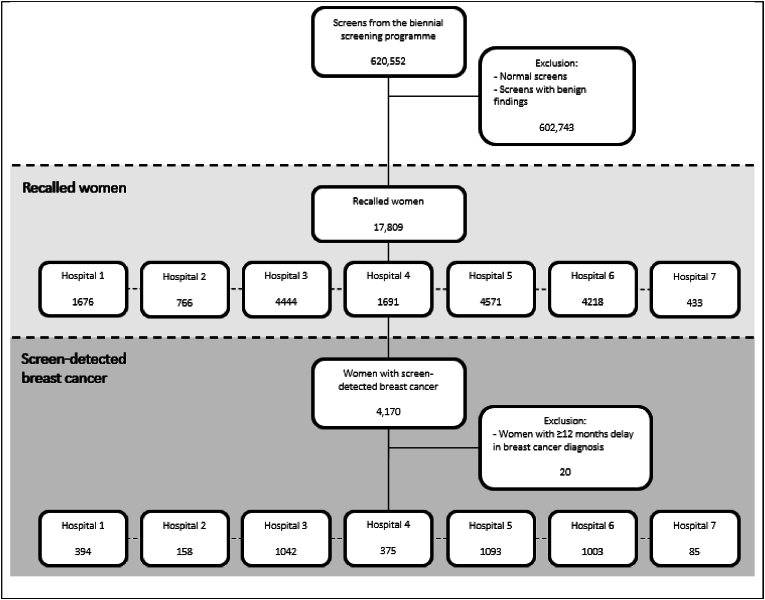
Flow-chart of the study population.

Fig. 1 shows a flow-chart of the study population. A total of 620,552 screening examinations had been performed, with 17,809 women recalled for a suspicious finding at their screening mammogram (recall rate 2.9 %). At clinical diagnostic work-up, breast cancer was diagnosed in 1470 of these recalled women, resulting in a cancer detection rate of 6.7 per 1000 screens. Twenty women with SDC were excluded due to a delayed breast cancer diagnosis of ≥12 months (range 12–97 months), resulting in a total of 1450 women with SDC included in this study.

Mean age at time of screening ranged from 61.9 to 63.6 years among the hospitals. Among the women with a SDC, the predominant BI-RADS classification at recall was BI-RADS 4 (“suspicious abnormality”), ranging from 47.7 % to 54.5 % among the hospitals (Table 1). Most women had no history of previous breast surgery (range 88.8–91.9 %).

**Table 1:** Patient and tumour characteristics of recalled women with screen-detected breast cancer per hospital.

Hospital	1	2	3	4	5	6	7
Patient characteristics							
Recalled women, No	394	158	1042	375	1093	1003	85
Mean age, y (range)	63.0 (50–75)	63.6 (50–75)	62.7 (50–75)	62.9 (50–75)	62.2 (50–76)	62.8 (50–75)	61.9 (50–75)
BI-RADS on screening mammography, % (No)							
0	13.7 % (54)	13.3 % (21)	12.6 % (131)	9.9 % (37)	11.9 % (130)	10.9 % (109)	17.6 % (15)
4	53.3 % (210)	48.7 % (77)	53.6 % (558)	52.8 % (198)	54.5 % (596)	47.7 % (478)	54.1 % (46)
5	17.8 % (70)	27.2 % (43)	17.5 % (182)	21.3 % (80)	19.5 % (213)	18.2 % (183)	16.5 % (14)
Unknown	15.2 % (60)	10.8 % (17)	16.4 % (171)	16.0 % (60)	14.1 % (154)	23.2 % (233)	11.8 % (10)
Previous breast surgery, % (No)							
No	89.8 % (354)	87.5 % (139)	91.9 % (958)	89.1 % (334)	89.0 % (973)	88.8 % (891)	89.4 % (76)
Cosmetic∗	2.0 % (8)	3.1 % (5)	1.8 % (19)	0.8 % (3)	1.6 % (18)	1.8 % (18)	2.4 % (2)
Other benign surgery	8.1 % (32)	8.1 % (13)	4.6 % (48)	9.3 % (35)	7.4 % (79)	6.9 % (69)	8.2 % (7)
BSC	–	0.6 % (1)	1.2 % (13)	0.8 % (3)	1.6 % (17)	1.7 % (17)	–
Mastectomy	–	–	0.4 % (4)	–	0.5 % (6)	0.8 % (8)	–
Tumour characteristics							
Histology, % (No)							
DCIS	19.8 % (78)	11.3 % (18)	23.5 % (245)	20.8 % (78)	20.1 % (220)	21.0 % (211)	29.4 % (25)
Invasive ductal	63.7 % (251)	70.0 % (110)	60.8 % (634)	60.5 % (227)	63.9 % (698)	61.8 % (620)	54.1 % (46)
Invasive lobular	9.1 % (36)	11.3 % (18)	8.4 % (88)	14.1 % (53)	10.0 % (109)	9.8 % (98)	9.4 % (8)
Invasive duct + lob	3.8 % (15)	0.6 % (1)	2.9 % (30)	–	0.9 % (10)	3.5 % (35)	2.4 % (2)
Invasive other	3.6 % (14)	6.9 % (11)	4.3 % (45)	4.5 % (17)	5.1 % (56)	3.9 % (39)	4.7 % (4)
Invasive tumours							
Mean size of invasive cancers, mm (range)	14.9 (1–85)	14.9 (2–45)	14.6 (1–120)	14.1 (1–80)	14.7 (1–90)	15.3 (1–77)	14.7 (3–49)
Invasive cancer size, % (No)o							
T1a-b	38.0 % (120)	27.5 % (39)	38.9 % (310)	36.0 % (107)	35.9 % (313)	33.5 % (265)	36.7 % (22)
T1c	40.5 % (128)	52.1 % (72)	43.3 % (345)	48.5 % (144)	45.4 % (396)	44.4 % (352)	40.0 % (24)
T2+	20.9 % (66)	19.7 % (28)	17.6 % (140)	15.2 % (45)	18.1 % (158)	21.7 % (172)	23.3 % (14)
Unknown	0.3 % (1)	–	0.1 % (1)	–	0.6 % (5)	0.4 % (3)	–
Lymph node status, No (%)							
N0	76.9 % (243)	78.9 % (110)	74.5 % (594)	79.8 % (237)	74.8 % (653)	72.7 % (576)	78.3 % (47)
N+	21.2 % (67)	19.0 % (27)	21.3 % (170)	19.5 % (58)	22.9 % (200)	19.4 % (154)	18.3 % (11)
Unknown	1.9 % (6)	2.1 % (3)	4.0 % (32)	0.7 % (2)	2.3 % (20)	7.8 % (62)	3.3 % (2)
Oestrogen receptor status, No (%)							
Positive	89.6 % (283)	88.0 % (123)	89.7 % (715)	88.6 % (263)	91.6 % (800)	90.0 % (713)	93.3 % (56)
Negative	9.8 % (31)	12.0 % (17)	9.5 % (76)	11.1 % (33)	7.9 % (69)	9.5 % (75)	6.7 % (4)
Unknown	0.6 % (2)	–	0.8 % (6)	0.3 % (1)	0.5 % (4)	0.5 % (4)	–
Progesterone receptor status, No (%)							
Positive	68.0 % (215)	71.8 % (100)	67.9 % (541)	69.7 % (207)	78.1 % (682)	69.6 % (551)	81.6 % (49)
Negative	31.3 % (99)	28.2 % (40)	31.4 % (250)	30.0 % (89)	21.4 % (187)	29.9 % (237)	18.3 % (11)
Unknown	0.6 % (2)	–	0.7 % (6)	0.3 % (1)	0.5 % (4)	0.5 % (4)	–
Her2Neu receptor status, No (%)							
Positive	10.8 % (34)	6.3 % (8)	8.8 % (70)	10.1 % (30)	7.6 % (66)	8.7 % (69)	5.0 % (3)
Negative	88.3 % (279)	93.0 % (131)	90.2 % (719)	89.6 % (266)	91.4 % (798)	90.7 % (718)	95.0 % (57)
Unknown	0.9 % (3)	0.7 % (1)	1.0 % (8)	0.3 % (1)	1.0 % (9)	0.6 % (5)	–
Bloom & Richardson score, % (No)							
1	41.8 % (132)	49.3 % (70)	46.3 % (369)	33.0 % (98)	48.0 % (419)	42.0 % (333)	46.7 % (28)
2	43.7 % (138)	35.2 % (49)	43.0 % (343)	52.2 % (155)	41.1 % (359)	45.8 % (363)	40.0 % (24)
3	13.9 % (44)	14.1 % (19)	9.7 % (77)	13.5 % (40)	9.0 % (79)	11.9 % (94)	13.3 % (8)
DCIS							
Mean size of DCIS, mm (range)	23.3 (2–90)	29.5 (2–114)	18.6 (2–130)	16.1 (2–85)	18.4 (1–100)	18.5 (1–125)	15.7 (2–52)
DCIS grade, % (No)							
Low	19.2 % (15)	11.1 % (2)	20.4 % (50)	15.4 % (12)	21.9 % (48)	16.1 % (34)	24.0 % (6)
Intermediate	32.1 % (25)	55.6 % (10)	34.3 % (84)	37.2 % (29)	39.3 % (86)	33.2 % (70)	48.0 % (12)
High	47.4 % (37)	33.3 % (6)	45.3 % (111)	47.4 % (37)	38.8 % (85)	50.7 % (107)	28.0 % (7)

∗ Cosmetic surgery includes augmentation and reduction. BI-RADS = Breast Imaging Reporting and Data System, BSC = breast-conserving surgery, DCIS = ductal carcinoma in situ.

### 3.2 Tumour characteristics

Tumour characteristics per hospital are displayed in Table 1. The hospitals with smaller numbers of breast cancers (hospital 2 and 7) showed some variation in the amount of DCIS (11.3 % and 29.4 %, respectively) and invasive ductal carcinomas (70.0 % and 54.1 %, respectively) when compared to the larger hospital groups (range 19.8–23.2 % and 60.5–63.9 %).

Smaller variations were found with respect to tumour size and proportions of invasive breast cancers with lymph node metastases. The mean size of invasive tumours ranged from 14.1 mm to 15.3 mm (p = 0.41). The hospitals showed comparable distributions in hormone receptor status of oestrogen, progesterone and HER2. Interhospital variation in the grade of the invasive cancers was limited, except for hospital 4 with 33.0 % grade 1 and 52.2 % grade 2, versus 41.8–49.3 % and 35.2–45.8 %, respectively, in other hospitals.

The mean DCIS size ranged from 15.7 mm to 29.5 mm, and mainly consisted of intermediate grade and high grade DCIS (range 76.0–88.9 %).

### 3.3 Work-up to treatment of screen-detected breast cancers

Almost all breast cancers had been pathologically confirmed by percutaneous biopsy prior to treatment (range 99.5–100 %). The proportions in the use of preoperative breast MRI differed among hospitals, ranging from 20.8 % to 35.8 % (p < 0.001, Table 2). Even after adjustment for case-mix, including age, breast tissue density of the screening mammogram, mammographic lesion type, T-stage, N-stage, Bloom & Richardson grading and histologic type, a significant difference between the hospitals persisted, with a smallest odds ratio (OR) of 0.35, 95 % CI 0.24–0.54 for hospital 6 versus hospital 1 (Table 3).

**Table 2:** Therapy characteristics of referred women with screen-detected breast cancer per hospital.

Hospital	1	2	3	4	5	6	7	P-value
Screen-detected breast cancer, No	394	158	1042	375	1093	1003	85	
Preoperative MRI, % (No)	35.8 % (141)	20.9 % (33)	20.8 % (217)	21.1 % (79)	24.7 % (270)	25.6 % (257)	27.1 % (23)	<0.001
Preoperative breast cancer confirmation, % (No)	99.5 % (392)	100 % (158)	99.8 % (1040)	100 % (375)	99.6 % (1089)	99.9 % (1002)	100 % (85)	n.a.
Invasive tumours								
Invasive cancers receiving NST, % (No)	10.1 % (32/316)	5.0 % (7/140)	13.3 % (106/797)	4.0 % (12/297)	5.2 % (45/873)	8.7 % (69/792)	8.3 % (5/60)	<0.001
Type of neoadjuvant therapy, % (No)								n.a.
Chemotherapy	90.6 % (29)	71.4 % (5)	69.8 % (74)	91.7 % (11)	82.2 % (37)	91.3 % (63)	80.0 % (4)	
Hormonal therapy	3.1 % (1)	14.3 % (1)	24.5 % (26)	8.3 % (1)	11.1 % (5)	2.9 % (2)	20.0 % (1)	
Chemo + immune	6.3 % (2)	–	3.8 % (4)	–	2.2 % (1)	2.9 % (2)	–	
Chemo + hormonal	–	–	1.9 % (2)	–	–	–	–	
Unknown	–	14.3 % (1)	–	–	4.4 % (2)	2.9 % (2)	–	
Final surgical treatment of invasive cancers, % (No)								<0.001∗
BCS	85.4 % (270)	87.1 % (122)	85.6 % (682)	79.5 % (236)	80.0 % (698)	81.9 % (649)	70.0 % (42)	
Mastectomy	12.7 % (40)	11.4 % (16)	13.4 % (107)	19.9 % (59)	19.4 % (169)	16.8 % (133)	28.3 % (17)	
Unknown/no surgery	0.9 % (3)	1.4 % (2)	1.4 % (11)	0.7 % (2)	0.7 % (6)	1.3 % (10)	1.7 % (1)	
Mean volume of resection specimen of invasive cancers treated by BCS, ml (range)	64 (7–268)	66 (14–251)	60 (6–265)	53 (2–348)	68 (4–442)	56 (6–369)	48 (11–130)	<0.001
Resection margins in case of primary BCS of invasive cancers								0.30
Negative	97.1 % (266)	95.9 % (118)	94.8 % (659)	95.9 % (231)	96.2 % (684)	94.3 % (623)	100 % (35)	
Positive	2.9 % (8)	4.1 % (5)	5.2 % (36)	4.1 % (10)	3.8 % (27)	5.7 % (38)	–	
DCIS								
Final surgical treatment of DCIS, % (No)								0.050∗
BCS	71.8 % (56)	61.1 % % (11)	82.2 % (199)	78.2 % (61)	77.7 % (171)	72.5 % (153)	76.0 % (19)	
Mastectomy	28.3 % (22)	38.9 % % (7)	16.1 % (39)	16.7 % (13)	18.6 % (41)	25.1 % (53)	24.0 % (6)	
Unknown/no surgery	–	–	2.5 % (6)	5.1 % (4)	3.6 % (8)	2.4 % (5)	–	
Mean volume of resection specimen of DCIS treated by BCS, ml (range)	58 (9–235)	52 (10–94)	55 (4–252)	43 (6–130)	57 (10–245)	46 (6–145)	46 (6–170)	0.06
Resection margins in case of primary BCS of DCIS, % (No)								0.21
Negative	94.8 % (55)	84.6 % (11)	95.6 % (196)	100 % (62)	95.4 % (167)	90.7 % (147)	100 % (20)	
Positive	5.2 % (3)	15.4 % (2)	4.4 % (9)	–	4.6 % (8)	9.3 % (15)	–	

∗ Excluding ‘Unknown/no surgery’. NST = neoadjuvant systemic therapy; DCIS = ductal carcinoma in-situ; MRI = magnetic resonance imaging; BCS = breast-conserving surgery, SD = Standard Deviation, n. a. = not applicable.

**Table 3:** Hospital variations in breast cancer treatment, multivariate analysis.

Hospital	Unadjusted OR (95 % CI)	P	Adjusted OR (95 % CI)	P
Preoperative MRI: yes vs. no∗ (women with SDC)				
1	Reference		Reference	
2	0.460 (0.294–0.720)	<0.001	0.373 (0.222–0.629)	<0.001
3	0.572 (0.444–0.737)	<0.001	0.480 (0.357–0.646)	<0.001
4	0.473 (0.365–0.613)	<0.001	0.419 (0.307–0.563)	<0.001
5	0.604 (0.468–0.779)	<0.001	0.536 (0.398–0.722)	<0.001
6	0.498 (0.359–0.692)	<0.001	0.354 (0.240–0.541)	<0.001
7	0.574 (0.330–0.996)	<0.05	0.501 (0.264–0.950)	<0.05
Neoadjuvant therapy: yes vs. no (women with invasive tumour)				
1	Reference			
2	0.519 (0.224–1.201)			
3	0.428 (0.265–0.692)	<0.001		
4	1.224 (0.808–1.854)			
5	0.819 (0.529–1.268)			
6	0.405 (0.209–0.748)	<0.01		
7	0.415 (0.124–1.388)			
Surgery: mastectomy vs. breast sparing surgery∗∗ (women with SDC)				
1	Reference		Reference	
2	1.049 (0.629–1.751)		0.710 (0.387–1.302)	
3	0.797 (0.584–1.087)		0.578 (0.395–0.845)	<0.01
4	1.142 (0.826–1.577)		0.908 (0.613–1.344)	
5	0.808 (0.590–1.106)		0.681 (0.464–0.999)	<0.05
6	0.775 (0.533–1.127)		0.460 (0.293–0.724)	<0.01
7	0.498 (0.287–0.865)	<0.05	0.285 (0.150–0.543)	<0.001
Surgery: mastectomy vs. breast sparing surgery∗∗∗ (women with invasive tumour)				
1			Reference	
2			0.732 (0.370–1.450)	
3			0.512 (0.331–0.793)	<0.01
4			0.802 (0.508–1.266)	
5			0.641 (0.409–1.004)	
6			0.378 (0.228–0.627)	<0.001
7			0.196 (0.094–0.409)	<0.001
Surgery: mastectomy vs. breast sparing surgery∗∗∗∗ (women with DCIS)				
1			Reference	
2			0.491 (0.111–2.172)	
3			1.050 (0.443–2.489)	
4			1.421 (0.613–3.296)	
5			0.608 (0.267–1.383)	
6			1.029 (0.330–3.203)	
7			0.852 (0.213–3.404)	
Free margins: yes vs. no (women with invasive tumour)				
1	Reference			
2	1.421 (0.457–4.424)			
3	1.269 (0.572–2.813)			
4	1.931 (0.891–4.188)			
5	2.172 (1.008–4.681)			
6	1.486 (0.589–3.748)			
7	0			

### 3.4 Neoadjuvant systemic therapy

Women had a significantly increased chance of receiving NST when treated in hospital 1 or 3 (range of NST use, 4.0–13.3 %, p < 0.001, Table 3). In all hospitals, the majority of women only received chemotherapy in the neoadjuvant setting (range 69.8–91.7 %). Correction for case-mix was not possible due to the small number of women receiving NST.

### 3.5 Type of breast surgery

Most women underwent BCS, which ranged from 70.0 % to 87.1 % for invasive breast cancers and from 61.1 % to 82.2 % for DCIS (Table 2). For patients with invasive breast cancers, we observed significant differences when comparing the type of surgery (BCS versus mastectomy) between the hospitals (p < 0.001) and these differences remained statistically significant after correction for confounders, with a smallest OR of 0.20, 95 % CI 0.09–0.41 for hospital 7 versus hospital 1 (Table 3). For patients with DCIS, the type of surgical treatment did not significantly differ between the hospitals after adjustment for case-mix.

A higher incidence of mastectomies was observed in patients in whom preoperative MRI was performed (Table 4); of those undergoing MRI 35.1 % underwent mastectomy (range 21.2%–47.8 %), compared to 11.7 % of those without preoperative MRI (range 7.8%–19.4 %) (p < 0.001).

**Table 4:** Proportions of surgical therapy with and without preoperative breast MRI.

Hospital	1	2	3	4	5	6	7	Total
Preoperative MRI performed								
BCS	68.1 % (96)	75.8 % (25)	62.7 % (138)	75.9 % (60)	58.5 % (159)	57.5 % (149)	47.8 % (11)	62.1 % (638)
Mastectomy	29.8 % (42)	21.2 % (7)	32.7 % (72)	22.8 % (18)	40.1 % (109)	39.0 % (101)	47.8 % (11)	35.1 % (360)
No Preoperative MRI performed								
BCS	92.2 % (235)	85.8 % (109)	89.9 % (744)	80.1 % (237)	86.6 % (717)	87.6 % (654)	80.6 % (50)	87.4 % (2746)
Mastectomy	7.8 % (20)	13.4 % (17)	9.2 % (76)	18.2 % (54)	12.2 % (101)	11.6 % (87)	19.4 % (12)	11.7 % (367)

MRI = magnetic resonance imaging; BCS = breast-conserving surgery.

The mean volume of the resection specimens of invasive tumours significantly differed between the hospitals, ranging from 48 ml to 68 ml (p < 0.001, Table 2). The mean volumes of the resection specimens of DCIS ranged from 43 to 58 ml (p = 0.06).

Both for invasive cancers and DCIS, most women had negative surgical resection margins after BCS and this outcome parameter was comparable for the hospitals. Resection margins were tumour positive in 0–5.7 % of the invasive cancers (p = 0.30), while DCIS showed 0–15.4 % positive resection margins (p = 0.21). Again, correction for confounders was not possible due to the small number of patients with positive surgical margins.

## 4 Discussion

In the current study we found statistically significant interhospital variation in the use of preoperative MRI, neoadjuvant systemic therapy (NST) and breast conserving surgery (BCS). In patients with invasive breast cancer treated by BCS, the mean volume of the resection specimens differed between hospitals. No variation was observed in preoperative breast cancer confirmation, and all hospitals showed low proportions of patients with positive resection margins in invasive tumours as well as DCIS. Our findings are consistent with previous studies from other European countries [6,14,15] and Australia [5,16], reporting on interhospital variation regarding therapeutic strategies in both symptomatic and asymptomatic women with breast cancer.

We observed significant interhospital variations in the use of preoperative breast MRI in women with - SDC (both invasive tumours and DCIS), even though the Dutch and European guidelines are quite clear regarding the indications for preoperative breast MRI (i.e., invasive lobular carcinoma and cases where the tumour size cannot be reliably assessed on conventional imaging) [[10], [11], [12], [9]]. Therefore, variation in the use of preoperative breast MRI could be a result of differences in interpretation of current guidelines, whether or not fueled by the rapid succession of new insights in breast cancer care.

Recent studies show varying results regarding the added value of preoperative breast MRI on the surgical planning. Invasive lobular cancer is one of the indications for breast MRI [[10], [11], [12], [9]], since the extent (including possible multifocal or bilateral tumours) of invasive lobular cancers is often underestimated by mammography and breast ultrasound [[17], [18], [19]]. The proportions of invasive lobular cancers among all invasive SDC varied from 10.9 % to 14.1 %. However, the hospitals with the highest proportions of invasive lobular cancer did not show the highest percentage of preoperative breast MRI (data not shown). Our multivariate analysis correcting for this and other possible confounders in the use of pre-operative MRI, also underlines that there must be other causes for the observed variation.

Multiple studies have examined the effect of preoperative MRI on surgical outcome, showing that its use is associated with a higher likelihood of mastectomy compared to women who do not undergo a preoperative MRI [[20], [21], [22], [23], [24], [25], [26], [27], [28]]. Our results are consistent with these studies. For example, the recent MIPA study showed significantly higher mastectomy rates (32,4 % versus 14,4 %) and lower reoperation rates (8.5 % versus 11,7 %) in women who underwent preoperative breast MRI compared to women who did not undergo preoperative breast MRI [29]. Moreover, preoperative breast MRI has been demonstrated to be associated with more (unilateral and bilateral) mastectomies and contralateral prophylactic mastectomies [30,31].

There have been substantial developments in the field of NST in the last decade. In the past, NST mainly consisted of chemotherapy, but recent studies have shown that hormone therapy and HER2-targeted therapy may also play an important role. For example, breast conservation could be achieved by neoadjuvant endocrine therapy in postmenopausal women with hormone receptor positive, HER-2 negative tumours [32]. Several studies report a significant increased use of NST, in women with both asymptomatic and symptomatic tumours [[33], [34], [35], [36], [37]]. These findings are likely of less importance in the screening population, with most SDC being small (T1) at diagnosis where NST is generally not indicated. We found significant interhospital variation in the use of NST, however, overall use of NST was low in our study population. These findings are supported by those of Vugts et al. (2016) reporting large interhospital variation in use of NST, and increased use of NST in the period 2003–2012 across all T-stages. However, only a small increase was found in T1 tumours (0.5 %–2.3 %) compared to T2 (2.7 %–27.0 %) and T3 tumours (30.6 %–70.9 %) [38].

We also observed significant interhospital variation in the use of BCS and mastectomy in the recalled women with SDC and this variation remained significant after correction for confounders. Our findings are in line with studies reporting on interhospital variations regarding surgical therapy in the Netherlands [39,40] and other countries. (**5,6,14–16**) These studies, comprising both screen-detected and symptomatic cancers, linked the variation to the accessibility of breast care [5,16], differences in guidelines [16], type of hospital [5,39] or socioeconomic differences [5,40]. In these studies, the type of hospital was subdivided in small versus large hospitals, teaching or non-teaching hospitals (concerning training surgical and/or internal medical residents), and general versus academic hospitals [5,39]. Our study comprised smaller and larger teaching hospitals and all but one of them were general hospitals. Though substantial differences in the access to health care are not expected in the Netherlands, Aarts et al. (2012) showed small but significant differences in the use of sentinel node biopsy, axillary lymph node dissection and BCS in women with breast cancer correlated to differences in patients’ socio-economic status (based on living area, income, employment and education) [40]. To our knowledge, there is only one other study reporting on interhospital variation in breast cancer surgery in the screening population. In 2012, Minicozzi et al. (2012) described variations in breast cancer treatment in Italy, where the regions with access to breast cancer screening showed higher proportions of early stage breast cancer and a higher change of receiving BCS and radiotherapy than other types of surgery. In addition to differences in availability of breast cancer screening, these findings may be due to a limited availability of radiotherapy facilities or lack of adherence to established guidelines by treating physicians [6]. Such regional differences are not expected to be present in the Netherlands as the Dutch screening programme is available nation-wide and there is comparable, good accessibility to radiation therapy throughout the country.

In breast cancer surgery, in addition to an optimal oncological surgical result, the cosmetic result is of great importance as well. In the case of BCS, the smallest possible excision volumes and large-scale use of oncoplastic techniques play an important role. The mean resection volumes in case of BCS differed between the hospitals. Remarkably, no differences were found in the proportions of free margins after surgery, which implies that certain hospitals may resect larger breast volumes than strictly necessary. Our outcomes are in line with those from a previous Dutch study presenting larger resection volumes than necessary in BCS [41]. The latter may be related to the experience of the surgeons, type and accuracy of preoperative tumour localisation [42], as well as expertise and availability of (onco)plastic surgeons.

The percentage of women with breast cancer, undergoing BCS versus mastectomy in a hospital population is used as a quality indicator by the European Commission Initiative on Breast Cancer (ECIBC) [5]. A threshold of 80 % is used for DCIS and 70 % for invasive tumours. However, this applies to both symptomatic and asymptomatic women. One might expect that the percentage of BCS could be even higher in the screening population, as these tumours are generally small at diagnosis.

Several national and international studies have been performed on interhospital variations regarding surgical therapy of breast cancer, but our study is the first that exclusively focuses on women with SDC. A larger database enabled us to obtain a high statistical power and perform multivariate analyses to correct for case-mix (with the exception of some small subgroups). Unfortunately, we lacked information about the socioeconomic status and comorbidities of patients, while these can vary between hospitals, which implies that we were only able to control for case mix to a limited extent. Small differences were observed in baseline characteristics, with some outliers in the smaller hospitals, though these are less important in the multivariate analysis. Adjuvant therapy was beyond the scope of the current study.

Interhospital variation does not necessarily have to be of clinical relevance. In our recently published study, focussing on interhospital variation in diagnostic work-up of SDC, the clinical impact appears to be marginal [43]. The variation observed in the current study adds to the overall interhospital variation in clinical care of women with screen detected breast cancer. However, more importantly, the most significant variations observed in this study, i.e. the difference in use of preoperative MRI and the difference in resection volume at BCS, are considered clinically relevant in itself. After all, the use of preoperative MRI is found to lead to a higher mastectomy rate and higher resection volumes at BCS may result in a less favourable cosmetic outcome. Both of which are not desirable. Moreover, as SDCs are generally small tumours, we should strive for higher proportions of women treated by BCS, preferably with low resection volumes.

Future studies could focus on survival outcome to determine the true impact of all observed variations. In the meantime, quality assurance of hospitals involved in the work-up and treatment of SDC may help to understand and decrease these differences, thereby improving breast cancer care.

## 5 Conclusion

Despite the presence of clear guidelines, we observed significant interhospital variation in surgical treatment of women with screen-detected breast cancer, specifically in use of preoperative MRI, type of surgery (mastectomy vs. BCS) and volume of resection specimen in BCS. Although these differences appear to be of clinical relevance, the impact on survival has yet to be determined.

Quality assurance in hospitals involved in the work-up and treatment of SDC may help to understand and decrease these differences, thereby improving breast cancer care. However, these results cannot be seen separately from the overall developments in breast cancer care. In these times where new evidence follows each other in rapid succession with the increasing need for personalised care, deviation from guidelines might sometimes be reasonable and acceptable.

## Funding

This research did not receive any specific grant from funding agencies in the public, commercial, or not-for-profit sectors.

## CRediT authorship contribution statement

**Eline L. van der Veer:** Writing – original draft, Visualization, Methodology, Investigation, Formal analysis, Data curation, Conceptualization. **Angela M.P. Coolen:** Writing – review & editing, Supervision, Methodology, Conceptualization. **Adriana M.J. Bluekens:** Writing – review & editing, Supervision, Methodology, Conceptualization. **Manon I. Generaal:** Methodology, Formal analysis, Data curation, Conceptualization. **Robert-Jan Schipper:** Writing – review & editing. **Wikke Setz-Pels:** Writing – review & editing. **Dominique J.P. van Uden:** Writing – review & editing. **Adri C. Voogd:** Writing – review & editing, Visualization, Supervision, Methodology, Formal analysis, Conceptualization. **Lucien E.M. Duijm:** Writing – review & editing, Visualization, Methodology, Investigation, Data curation, Conceptualization.

## Availability of data and materials

The data that support the findings of this study are available from the corresponding author upon reasonable request.

## Ethical approval

Ethical approval was not required for the current study, according to the Dutch Central Committee on Research involving Human Subjects (CCMO).

∗Adjusted OR; adjusted for hospital group, age, breast tissue density on screening mammogram, type of lesion, T-stage, N-stage, Bloom & Richardson score and histology. ∗∗Adjusted OR; adjusted for hospital group, breast tissue density on screening mammogram, type of lesion, preoperative MRI, free margins, tumour size (in mm), previous surgery, Bloom & Richardson score and histology. ∗∗∗Adjusted OR; adjusted for hospital group, type of lesion, preoperative MRI, free margins, tumour size (in mm), previous surgery and N-stage. ∗∗∗∗ Adjusted OR; adjusted for breast tissue density on screening mammogram, preoperative MRI, tumour size (in mm) and previous surgery.

## Funding information

This research did not receive any specific grant from funding agencies in the public, commercial, or not-for-profit sectors.

## Declaration of competing interest

The authors declare that they have no known competing financial interests or personal relationships that could have appeared to influence the work reported in this paper.
